# Evaluations of maxillary sinus pathologies using CBCT imaging

**DOI:** 10.6026/973206300223590

**Published:** 2026-06-30

**Authors:** Gomati Dhande, Rajkumari Gupta, Prarthana G.A, Srinivas Murthy S.T., Dhanuja Rani J., Tarang K. Mehta, Miral Mehta

**Affiliations:** 1 Department of Dentistry, ShriJawahir Hospital, Jaisalmer Medical College, Jaisalmer, Rajasthan, India; 2 Department of Oral Pathology, Vyas Dental College and Hospital, Jodhpur, Rajasthan, India; 3 Department of Oral Medicine and Radiology, Rajarajeswari Dental College and Hospital, Bengaluru, Karnataka, India; 4 Department of Pedodontics and Preventive Dentistry, Government Dental College and Research Institute, Ballari, Karnataka, India; 5 Department of Oral and Maxillofacial Pathology, Government Dental College and Research Institute, Ballari, Karnataka, India; 6 Department of Oral & Maxillofacial Pathology and Oral Microbiology, K M Shah Dental College & Hospital, Sumandeep Vidyapeeth Deemed to University, Vadodara, Gujarat, India; 7 Department of Pedodontics and Preventive Dentistry, Karnavati School of Dentistry, Karnavati University, Gandhinagar, Gujarat, India

**Keywords:** Cone-beam computed tomography (CBCT), maxillary sinus, sinus pathology, mucosal thickening, anatomical variations, diagnostic imaging, dental radiology

## Abstract

The detection of maxillary sinus pathologies is often challenging due to their asymptomatic nature and the limitations of conventional imaging techniques. Therefore, it is of interest to use cone-beam computed tomography (CBCT) to examine maxillary sinus issues. They reviewed 120 CBCT scans to identify both disease and anatomical differences. Mucosal thickening was the most common finding, followed by sinus opacification and retention cysts. These issues appeared more often in older patients. Thus, CBCT proved to be a reliable way to detect sinus problems and can support better clinical decisions.

## Background:

Maxillary sinus pathologies are common in clinical practice and are often found by chance during radiographic exams [1]. Traditional imaging methods have limits because they only show two-dimensional views and cannot fully assess sinus anatomy [2]. Cone-beam computed tomography (CBCT) has improved how we evaluate the maxillary sinus by offering high-resolution, three-dimensional images [2, 4]. This technology helps accurately assess mucosal changes, anatomical differences and sinus pneumatization, which are important for diagnosis and treatment planning [3]. Several studies have found that incidental sinus findings are common on CBCT scans, highlighting the need for thorough radiographic interpretation [4, 5]. CBCT is also important for planning procedures like implant placement and sinus floor elevation, as it allows for precise measurement of bone volume and anatomical structures [4, 6]. Assessing vascular anatomy and sinus wall thickness with CBCT can help lower surgical risks and improve patient outcomes [6, 7]. Recent developments stress the need to use CBCT wisely, following principles like ALADAIP to balance diagnostic benefits with radiation exposure [8, 9]. The differences in interpretation remain, showing the need for standardized diagnostic methods [1, 10]. Therefore, it is of interest to evaluate maxillary sinus pathologies using CBCT and to assess its value in everyday dental practice.

## Materials and Methods:

This retrospective study looked at maxillary sinus problems using cone-beam computed tomography (CBCT). Researchers chose 120 patient scans from the radiology database based on specific criteria. Only scans with clear images and full views of the maxillary sinus were included. Scans with artifacts, past surgery, or missing records were left out. All scans used the same CBCT settings to keep image quality the same. The images were reviewed in three planes using special software. Researchers checked for mucosal thickening, sinus opacification, retention cysts, polyps and differences like sinus pneumatization. Two experienced oral radiologists reviewed the images separately to avoid bias and any disagreements were discussed until they agreed. All results were recorded and grouped for analysis. The frequency of each sinus problem was calculated using basic statistics. The study followed ethical rules and patient privacy was always protected.

## Results:

In total, 120 chloride-based (CBCT) mechanisms were evaluated for the presence of maxillary sinus pathologies and anatomical variations. Of the 120 scans, palpable pathologic findings were seen on a CT scan in at least one case (68.3%, N = 82). In contrast, only 26 cases (31.7%) showed no evidence of sinus pathology on CT imaging, as enumerated below and summarised in Table 2. Our data demonstrate that sinus abnormalities of the maxilla are highly prevalent in our population and strengthen the diagnostic basis for the potential unrecognizability of sinus disease because it is clinically asymptomatic. The distribution of various maxillary sinus pathologies is described in Table 1. The most common finding was mucosal thickening, found in 54 cases (45.0%). Among them, sinus opacification was seen in 28 cases (23.3%), and retention cysts were found in 22 cases (18.3%). Ten cases (8.3%) were polyps, and 6 findings were in the miscellaneous category, representing about the same amount (5%) (Table 1). The thickening of the mucosa in most cases points to inflammatory changes (mostly pathological finding-sinusitis) of the sinusimalaboriosa. Table 2 shows the anatomical variations and sinus involvement pattern. Sinus pneumatization was present in 40 cases (33.3%), indicating a significant frequency of anatomical expansion of the sinus cavity into surrounding alveolar bone. Forty-six patients (38.3%) had unilateral sinus involvement, and 36 patients (30.0%) had bilateral disease, as summarised in Table 2. The marginally higher prevalence of unilateral pathology may reflect localised odontogenic or inflammatory etiologies involving an isolated sinus cavity.The sinus pathologies by gender are shown in Table 3. Of the 68 male patients, pathological findings were present in 48 cases (70.6%); however, no pathology was noted for an additional 20 cases (29.4%). Of 52 cases with these findings in females, 34 (65.4%) showed sinus abnormality; one of the normal cases was a cystic lesion, and the others were negative on CT scan. Postnasal drip was noted among all patients, regardless of gender, as a probable cause of paranasal sinus involvement seen on dot-scan (Table 3). The occurrence of sinus pathologies was slightly higher in males. Still, the difference between gender groups did not reach statistical significance, indicating that there is probably no major effect of gender on maxillary sinus diseases. The severity of mucosal thickening was also analysed in 54 affected cases (Table 4). Mild mucosal thickening [6mm in 14(25.9 %) Cases (Table 4). The more frequent mild mucosal thickening indicates that even if CBCT can detect early inflammatory changes before sinus disease clinically becomes advanced.Age Distribution of maxillary sinus pathologies is shown in Table 5. Of 30 patients with sinus pathology, 18 were in the 18-30 years age group (53.3%). Pathological findings were observed in 30 of the total 42 cases (71.4%) patients who referred to our clinic, and whose histopathologic finding was correlated with age for groups: women aged under thirty years; this prevalence increased significantly among those aged between 31-45 location group from where pathological findings are presented at variable sites, however vivid specimens by imaging modalities possibly corresponding clinical tendonopathy appeared a bit dimly widespread had been reported elsewhere. Most men were aged 46-60 years (n=28,78.6%) and the highest prevalence was among those in the latter age ranges; with,22 out of these 28 patients seen at this range.Table 6 demonstrates the relationship between mucosal thickening and age group. Mucosal thickening was observed in 10 of 30 patients (33.3%) aged 18 to 30 years. At the same time, this ratio increased to as high as 42% for the age group of ≥ 66 years, comprising more than half of those over 60, taggingsignificant comorbidities with clinical morphology descriptor score upper age bracket(12-roots). The top of the mucosal thickening becomes more common with age, further supporting the chance that cumulative inflammatory changes are underway. In conclusion, the findings of the present study show that CBCT is highly effective for detecting maxillary sinus pathology and anatomical variations. CBCT provides a Uniform Three-Dimensional view of inflammatory mucosal changes and sinus opacification, as well as retention cyst (RC) features associated with the maxillary sinuses. Also, help in preoperative planning to assess the amount or presence of IP before performing dental treatments.

## Discussion:

This study found that maxillary sinus problems are common on CBCT scans, with mucosal thickening being the most frequent. These results agree with earlier research showing that inflammation is the most common sinus issue seen in imaging [11, 12]. The frequent discovery of unexpected findings highlights the need for careful CBCT review, even in patients without symptoms. CBCT is widely accepted as a reliable way to check maxillary sinus anatomy and disease because it gives clear three-dimensional images. Other studies have shown that CBCT can detect subtle changes like mucosal thickening, retention cysts and sinus opacification that standard imaging might miss [13, 14]. Our results support this, as we found a wide range of sinus problems. We also saw that anatomical differences, like sinus pneumatization, were common. Previous research has found similar results, noting that these differences can affect implant planning and surgery [15, 16]. This shows why pre-operative CBCT scans are important to help avoid complications. When we looked at gender, we found no major difference in sinus problems, which matches other studies [17]. However, sinus issues increased with age, likely due to longer exposure to health and environmental factors. Most mucosal thickening cases were mild, suggesting that early inflammation is being found more often, probably because of better imaging like CBCT. Other studies have seen the same pattern [18]. Our findings also show that CBCT helps improve diagnosis and clinical decisions. Guidelines recommend using CBCT properly to get the most benefit while keeping radiation exposure low [19]. Imaging of the paranasal sinuses can also reveal findings that may change patient care [20]. Nascimento *et al.* evaluated maxillary sinus pathologies using Cone Beam Computed Tomography (CBCT) and reported that mucosal thickening was the most common radiographic finding. The authors emphasized that CBCT provides accurate three-dimensional visualization for detecting inflammatory sinus changes and anatomical variations that may not be clearly visible on conventional radiographs. Their findings highlighted the importance of CBCT in diagnosing maxillary sinus abnormalities and improving treatment planning in dental practice [21]. Despite its benefits, CBCT has some limits, such as differences in how scans are read and the risk of over diagnosing minor findings [22, 23, 24]. It is important to match scan results with clinical information to avoid unnecessary treatments [25, 26]. More research with larger groups and follow-up is needed to confirm these results. Overall, our study shows that CBCT is important for finding maxillary sinus problems and anatomical differences. Using detailed imaging along with clinical assessment can improve diagnosis and help plan better dental treatments.

## Conclusion:

CBCT imaging was highly effective at detecting maxillary sinus problems and anatomical differences. Mucosal thickening was the most common finding and it became more frequent with age. Thus, CBCT is a valuable tool for accurate diagnosis and better clinical decisions in dental practice.

## Advancement of knowledge:

This study shows that maxillary sinus problems are common and often found with CBCT, even in patients without symptoms. CBCT can spot both disease and anatomical differences more accurately than older methods. These findings can help improve diagnosis and support using CBCT as a reliable tool for treatment planning.

## Figures and Tables

**Table 1:** Distribution of maxillary sinus pathologies(n = 120)

**Maxillary Sinus Pathologies**	**n**	**%**
Mucosal thickening	54	45.00%
Sinus opacification	28	23.30%
Retention cyst	22	18.30%
Polyps	10	8.30%
Other findings	6	5.00%
Total	120	100%

**Table 2:** Distribution of anatomical variations and sinus involvement

**Findings**	**Number of Cases(n)**	**Percentage (%)**
Sinus pneumatization	40	33.3
Unilateral involvement	46	38.3
Bilateral involvement	36	30
No pathology	38	31.7

**Table 3:** Distribution of sinus pathologies based on gender

**Gender**	**Total Cases(n)**	**Pathology Present**	**No Pathology**	**Percentage With Pathology (%)**
Male	68	48	20	70.6
Female	52	34	18	65.4
Total	120	82	38	68.3

**Table 4:** Severity of mucosal thickening in maxillary sinus (n = 54)

**Severity of Mucosal Thickening**	**Number of Cases (n)**	**Percentage (%)**
Mild (<3 mm)	22	40.7
Moderate (3-6 mm)	18	33.3
Severe (>6 mm)	14	25.9
Total	54	100

**Table 5:** Age-wise distribution of maxillary sinus pathologies

**Age Group (Years)**	**Total Cases (n)**	**Cases with Pathology**	**Percentage with Pathology (%) **
18-30	30	16	53.3
31-45	42	30	71.4
46-60	28	22	78.6
>60	20	14	70
Total	120	82	68.3

**Table 6:** Association between mucosal thickening and age group

**Age Group (Years)**	**Cases with Mucosal Thickening**	**Total Cases (n)**	**Percentage (%)**
18-30	10	30	33.3
31-45	18	42	42.8
46-60	16	28	57.1
>60	10	20	50
